# Multiple Scattering-Enhanced Fluorescence Within Randomly Oriented Low-Index Polymer Nanofiber Sensors

**DOI:** 10.3390/bios15020097

**Published:** 2025-02-08

**Authors:** Jing Sun, Tao Huang, Zhongyang Wang

**Affiliations:** 1 Shanghai Advanced Research Institute, Chinese Academy of Sciences, Shanghai 201210, China; sunj@sari.ac.cn; 2 University of Chinese Academy of Sciences, Beijing 100049, China; 3 Department of Materials Science and Engineering, Southern University of Science and Technology, Shenzhen 518055, China

**Keywords:** multiple scattering, surface-enhanced fluorescence, randomly oriented, low-index nanofiber sensor

## Abstract

Fluorescence enhancement technologies play a crucial role in biological and chemical sensors. Currently, effective fluorescence sensors primarily rely on noble metals and high-index dielectric nanostructures. While effective, they are plagued by optical losses and complex fabrication processes. In contrast, low-index material nanostructures offer significant advantages, including the absence of optical losses, ease of fabrication, and cost-effectiveness, but they face the challenge of weaker electric field enhancement. Here, we designed a low-index, randomly oriented polyvinyl acetate (PVAc) nanofiber sensor via scalable electrospinning, enabling multiple scattering within the disordered nanofibers and resulting in an impressive surface-enhanced fluorescence factor of 1170. This sensor achieves a detection limit for rhodamine 6G as low as 7.24 fM, outperforming the reported fluorescence biosensors. Further results of photoluminescence decay dynamics and random lasing validate the effectiveness of multiple scattering in enhancing fluorescence within the polymer nanofiber sensor. With its excellent performance and scalable production process, this randomly oriented, low-index polymer nanofiber sensor offers a promising new pathway for efficient surface-enhanced fluorescence based on multiple scattering. Furthermore, PVAc nanofibers can be extended to other low-index materials capable of forming randomly oriented nanostructures, offering significant potential for cost-effective, high-performance fluorescence sensor applications.

## 1. Introduction

Surface-enhanced fluorescence (SEF) and surface-enhanced Raman scattering (SERS) are crucial optical detection technologies for chemical sensors and biosensors. Noble metal nanostructure sensors provide efficient means for SEF and SERS through plasmonic effects or nanogaps [1]. However, these sensors face significant challenges due to strong optical losses. To achieve subwavelength plasmonic resonators, part of the optical energy is converted into the kinetic energy of electrons [2], which results in Joule heating. Although photothermal effects are advantageous in several applications, including cancer therapy [3], imaging [4] and catalytic process [5], the resultant local heating can damage biomolecules and the nanostructure themselves, limiting their practical use in biosensors. Meanwhile, nonradiative decay channels induced by optical losses typically result in low fluorescence efficiency [6,7]. Consequently, SEF and SERS are generally incompatible within the same nanostructure. The SEF enhancement factor (F_SEF_) usually does not exceed 10^3^ [8], while the SERS enhancement factor can reach over 10^11^ [9], making SERS a more widely applied technique in plasmonic sensors. It is well established that fluorescence is sharply enhanced within about 10 nm of a surface, but it is strongly quenched within about 5 nm [10]. Therefore, achieving significant fluorescence enhancement requires precise control over the critical distance, which requires a complicated fabrication process and still cannot resolve these intrinsic losses in the plasmonic sensor.

In this context, high-index dielectric materials such as crystalline silicon, germanium, and gallium phosphide have been developed for all-dielectric sensors [11,12,13]. These sensors can achieve the plasmonic-like enhancement effect while overcoming the issues of plasmonic sensors, particularly by mitigating fluorescence quenching effects [14]. But one efficient all-dielectric sensor requires the following: the size of the nanostructure cell in 100–200 nm to promise that the lowest order Mie resonances appear in the visible region [15,16] and forming nanogaps by combining two or several nanostructures to obtain mode hybridization and electromagnetic field enhancement [17]. Complicated nanostructures such as nanodisks, nanopillars, nanorods, and nanorings benefit from advanced nanofabrication technologies, like electron beam lithography in the semiconductor industry [18]. Optimized designs have demonstrated the F_SEF_ up to 10^3^ [19]. Precise control over nanostructure size, geometry, spatial arrangement, and reproducibility is critical for advancing fundamental research. However, it has a high cost and low yield for practical applications. Moreover, high-index materials like Si are transparent in the near-infrared range; they still absorb light in the visible range, leading to increased ohmic losses and reduced performance as dielectric resonators in this range [18].

So widely available low-index dielectric materials such as metal oxides and polymers, which exhibit near-zero losses in the visible range, have become increasingly attractive. Although TiO_2_ [20], ZnO [21], and SiO_2_ [22] have attracted the most attention, these materials generally exhibit weak SEF with an enhancement factor of around 10^2^, which is insufficient for fluorescence-based sensor applications. A notable exception is a TiO_2_ film with a nanogap-rich structure, which achieves an F_SEF_ of 2000 based on the electromagnetic field enhancement [23]. However, it is still infrequent for the low-index material-based SEF. Extremely transparent and flexible polymer materials, which are easy to process and offer tunable optical properties, have been widely used in nanophotonic devices and techniques [24], such as polyvinyl alcohol (PVA) nanofiber-based pH [25], humidity and temperature sensors [26], and random lasing [27,28]. Specifically, polymer-based disordered scattering media have been employed in random lasers [29], inspiring the idea that the disordered polymer media could be potential candidates for fluorescence enhancement via multiple scattering. Additionally, polymer nanostructure–metal hybrid systems have also produced large field enhancements [30] although noble metals still play a nonnegligible role in providing strong electric field enhancement. However, all-dielectric, low-index polymer nanostructures for SEF have not been fully realized in the visible range.

Herein, we present a simple and efficient strategy for the direct preparation of flexible, all-dielectric, low-index, randomly oriented poly (vinyl acetate) (PVAc) nanofibers for SEF. The polymer nanofiber-based fluorescence sensor was produced using a scalable electrostatic spinning process that naturally forms complex and disordered nanofiber patterns. The light could be transported, scattered, and amplified within the disordered nanofibers, known as multiple scattering. Random lasing induced by multiple scattering within the PVAc nanofibers exhibits a laser mode with a linewidth of 0.24 nm and a quality factor of 2270. The corresponding transport mean free path $l_{t}$ is estimated in the range of 2.5–4.5 μm. As a result, using rhodamine 6G as a probe molecule, the average PL enhancement factor (F_SEF_) was experimentally measured at an impressive 1170. For all these performances, this efficient sensor achieves a limit of detection (LoD) of 7.24 fM for the probe molecule. For the detection of biomolecules, a Cy3-labeled DNA aptamer was utilized as a detection target, exhibiting a large linear dynamic range from 0.1 to 500 nM, along with a limit of detection of 7.6 nM. Furthermore, decay kinetics results prove that the sensor maintains the intrinsic radiation rate and fluorescence lifetime of the probe molecule, ensuring no damage to the detection target—an essential feature for biosensors.

In addition, the polymer nanofiber sensor does not require critical design or precise control over the target–sensor distance, which is indispensable for the metallic counterparts to avoid fluorescence quenching. This research achieves a substantial SEF effect in low-index, all-polymer nanofiber fluorescence sensors. Due to the universality of multiple scattering, the strategy employed for PVAc can be extended to other polymers or low-index dielectric materials that can be fabricated in disordered scattering media. This work paves the way for the development and application of more low-index, nanostructure-based multiple scattering-enhanced fluorescence sensors.

## 2. Materials and Methods

### 2.1. Fabrication of PVAc Nanofiber Film

The electrospinning process is described as follows. The solution of 25 wt.% PVAc was prepared by intermittently adding PVAc powder to the mixture of N, N′-dimethylformamide (DMF), and ethanoic acid. The mixture was stirred for 3 h at room temperature in order to obtain homogeneously dissolved solutions. The resulting PVAc solution was electrospun under a voltage of 15 kV, a spinning distance of 15 cm, a feeding rate of 0.8 mL h^−1^, and a 1 mm needle tip. Commercially available aluminum foil was employed as the collector electrode. The PVAc nanofiber film was obtained with a thickness of 20 μm for 2 h. In the same way, the samples with different thicknesses were fabricated by controlling the spinning time.

### 2.2. Microscopy and Optical Measurement

The PL spectra and fluorescence image were measured using a homemade micro-photoluminescence setup on a conventional inverted microscope (IX83, Olympus, Tokyo, Japan) with a microscope objective (20×, NA: 0.4, Olympus) and a continuous-wave laser at 532 nm (OBIS-532 nm-LS-80 mW, Coherent, Saxonburg, PA, USA). The measurement of PL spectra or fluorescent images was controlled by a removable lens (L1) and a removable mirror (M1). Without L1 and M1, the pump light was focused on the sample through the microscope objective and the emission light was collected with the same objective, followed by a bandpass emission filter (ZET532, Chroma, Taiwan, China) and guided to the spectrograph (IHR550, HORRIBA, Kyoto, Japan) with a grating of 300 mm^−1^ and an InGaAs photodetector. With L1 and M1 in the setup, the wide-field fluorescence image collected from the same local sample was detected using a scientific CMOS (Prime 95B, Photometrics, Tucson, AZ, USA). In fluorescence lifetime experiments, the time-correlated single photon counting (HydraHarp 400, Pico Quant, Berlin, Germany) was measured by a Ti: sapphire system (Chameleon Vision, Coherent) with a repetition rate of 80 MHz, excited by a 530 nm laser, and a single-photon detector (PMA 185, PicoQuant, Berlin, Germany). The random lasing was pumped by a DPSS Q-switched pulsed Nd: YAG laser with 532 nm and 10 ns pulse duration (NANO-DPSS, Litron Lasers, Rugby, UK). Reflectance spectra were measured using a white light generated by the halogen light source.

### 2.3. Preparation for Fluorescence Measurements

The fluorescent dye (rhodamine 6G, R6G, Sigma Aldrich, St. Louis, MO, USA) aqueous solution was prepared with different concentrations. For fluorescence enhancement measurements, 10 μL of 10^−9^ M R6G aqueous solution was dropped onto SiO_2_/Si, Al foil, and PVAc nanofiber film of different thicknesses and air-dried. For random lasing, a 10^−3^ M R6G aqueous solution was used. For assessment of the detection limit, 10 μL of R6G aqueous solution with concentrations of 10^−10^ M to 10^−18^ M was dropped onto the PVAc nanofiber film, respectively. A 2 mm circle area was formed on PVAc nanofiber film after air-drying (3.14 mm^2^). For the biomolecular detection, a Cy3-labeled DNA aptamer (aptamer-Cy3, DNA sequence: Cy3-CAC TAC AGA GGT TGC GTC TGT CCC ACG TTG TCA TGG GGG GTT GGC CTG TTT TTT TTT TTG AGC CAC TGG ATA C) was obtained from Sangon Biotech (Shanghai) Co., Ltd., (Shanghai, China) and diluted to various concentrations ranging from 0.1 nM to 2000 nM in phosphate-buffered saline (PBS) buffer. Before use, in order to adsorb aptamer-Cy3, the PVAc nanofiber film was treated with oxygen plasma for 30 s to increase its hydrophilicity. Then 20 µL of aptamer-Cy3 of the desired concentration was deposited onto the PVAc nanofiber film for 5 min. Following the aptamer-Cy3 deposition, the samples were rinsed with deionized water gently to remove the salt and unfixed DNA molecules and then air-dried. Prior to all the experiments, the tools and containers were rinsed with ethanol and exposed to UV ozone treatment for 1 min to remove any possible organic impurities.

### 2.4. Finite-Difference Time-Domain (FDTD) Simulations

A commercial software (FDTD solution (2023 R2.3), Lumerical (Vancouver, BC, Canada)) was used to calculate the electric field distribution of the PVAc nanofibers at an excitation wavelength of 532 nm. The light was irradiated as a plane wave. The refractive index of PVAc was used as n = 1.46. The size of the nanofiber was estimated by SEM. We set perfectly matched layer boundary conditions for the z direction and Bloch boundary conditions for the x and y directions of the simulation region with a single unit. The field profiles were obtained from the DFT monitors.

## 3. Results and Discussion

### 3.1. Multiple Scattering-Enhanced Fluorescence Strategy

**Figure 1:**
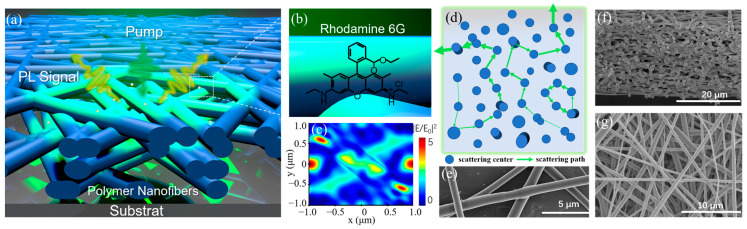
Schematic illustration of multiple scattering-enhanced fluorescence. (**a**) Illustration of the concept of using polymer nanofiber for surface-enhanced fluorescence; the yellowish green dot is the Rhodamine 6G molecular probe (R6G). (**b**) The enlarged surface state of the white dash box in (**a**) and the structural formula of R6G. (**c**) The FDTD-simulated electric field intensity distribution of two crossed nanofibers with the refractive index of 1.46 for incident plane waves at 532 nm. (**d**) The model for a multiple scattering path of the PL signal within the polymer nanofibers on the cross section. Multiple scattering increases the path length or forms a closed loop path of the PL signal inside the gain medium. Scanning electron micrographs (SEM) of polymer nanofibers with an average diameter of 550 nm: (**e**) the enlarged crossed nanofibers; (**f**) the cross section; (**g**) surface topography.

Overlapping and randomly oriented polymer nanofibers serve as an effective random scattering medium and have been utilized in random lasers due to their network topology and multiple scattering effects [29]. Our strategy leverages the efficient multiple scattering of disordered polymer nanofibers to detect low-concentration fluorescence probes, as shown schematically in Figure 1a–d. Using all-dielectric polymer nanofibers as an optical sensor, the issue of fluorescence quenching is circumvented, and the weak photoluminescence (PL) signal from the low-concentration fluorescence probes is enhanced in two steps. Firstly, the PL signal is enhanced by the local electric field around polymer nanofibers. The simulated electric field intensity distribution for two intersecting nanofibers, obtained via FDTD simulations, is shown in Figure 1c (more details are given in Figure S1, including the electric field intensity of the cross section of intersecting nanofibers, single nanofiber, vertically parallel nanofibers, and ring-shaped nanofiber). Due to the low refractive index of polymer, near electric fields around the polymer nanofibers provide a weak enhancement of 3–8 times (|E/E_0_|^2^ = 3–8), which is relatively low compared to the enhancements observed with noble metals and high-index dielectric materials. Secondly, because of the refractive index difference between polymer nanofibers and air, the PL signal enhanced by the electric field undergoes multiple scattering between the polymer nanofibers again. This process is similar to light being reflected multiple times within a laser cavity to generate strong optical feedback. The potential scattering paths are shown in Figure 1d. By increasing the path length or forming closed-loop scattering paths, the PL signal is amplified and eventually becomes detectable.

To achieve the desired nanofiber structure for SEF, we fabricated PVAc nanofiber film on Al foil through electrospinning. This approach offers a significant advantage in producing complex and disordered nanofiber patterns (scanning electron micrographs (SEM) in Figure 1e–g). The cross section of nanofiber film reveals a multilayered random stack structure as expected (Figure 1f). The obtained PVAc nanofiber film is composed of cross-linked subwavelength nanofibers with an average diameter centered around 550 nm (Figure 1g and Figure S2). A typical PVAc nanofiber film is white in appearance and measures 30 cm in length, 10 cm in width, and 20 μm in thickness (Figure S2). The white appearance indicates strong multiple scattering of visible light within the multilayered structure of randomly oriented and stacked nanofibers [31].

### 3.2. Polymer Nanofiber-Based F_SEF_

**Figure 2:**
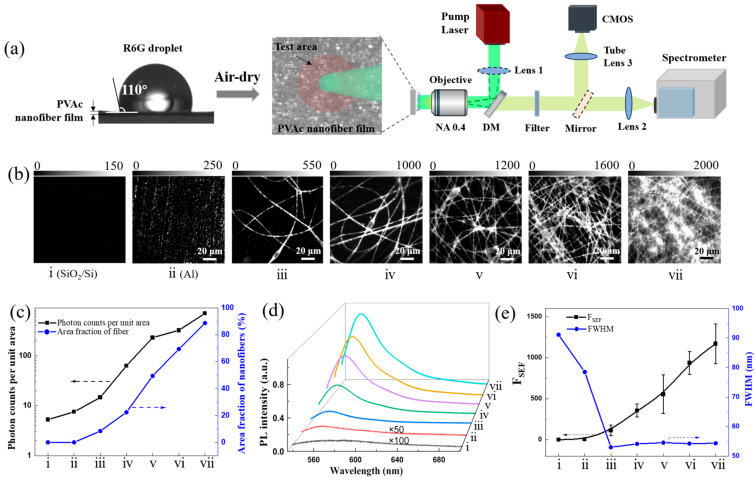
SEF of all-dielectric polymer nanofibers. (**a**) Left: The contact angle of R6G solution on PVAc nanofiber film. Middle: The top view of evaporated R6G solution on film; the marked pink area represents the test area. Right: The schematic diagram of the home-built optical microscope in reflection configuration with spectra system. Lens 1 and mirror: removable lens and mirror; DM: dichroic mirror; filter: 532 nm bandpass filter. (**b**) The fluorescence images of R6G on the control of i (SiO_2_/Si) and ii (Al foil), on the nanofibers with increasing density and thickness, iii (a few nanofibers), iv (dozens of nanofibers), v (layer of nanofibers), vi (a few layers), vii (dozens of layers with a thickness of ~20 μm). (**c**) The photon counts per unit area and area fraction of nanofibers obtained from the images in (**b**) through Image J. (**d**) The PL spectra of R6G at the location in (**b**). (**e**) The F_SEF_ and the full width at half maximum (FWHM) obtained from the PL spectra in (**d**).

The fluorescence enhancement of R6G on disordered PVAc nanofiber film was performed using a home-built reflected fluorescence microscopy equipped with a spectra system, based on prior work [32], as shown in Figure 2a. Fluorescence emission was collected in a backscattering configuration, using a bandpass filter to remove the contribution from the excitation laser in the spectrum. Both microscopic images and PL spectra were recorded from the same area. To experimentally evaluate the F_SEF_, PVAc nanofibers with increasing density and thickness were fabricated on the Al foil. The R6G solution at the same concentration was dropped on the nanofibers and air-dried, respectively. Meanwhile, the SiO_2_/Si and the Al substrate were used as the control samples. The PVAc nanofiber film exhibited hydrophobic properties, with a contact angle of 110° (Figure 2a).

As shown in Figure 2b, fluorescence images became brighter as the density and thickness of the PVAc nanofibers increased, compared to the control samples. The randomly orientated nanofibers are clearly visible in the fluorescence images (the corresponding optical images are given in Figure S3). To quantify the PL intensity in the fluorescence images for samples i–vii, we counted photon counts per unit area and nanofiber area fraction using Image J (https://imagej.net) (Figure 2c). For the control sample ii (Al), the photon counts per unit area are higher than sample i (SiO_2_/Si) due to the surface plasmon effect of Al [33]. As the density and thickness of PVAc nanofibers increased, the nanofiber area fraction grew from 0% to ~90% (100% for sample vii based on SEM), and the photon counts per unit area increased from ~5 to ~700 counts. The spectra in Figure 2d were obtained from the locations in Figure 2b. The PL intensity significantly increased, showing the same trend as the photon counts per unit area for sample i–vii. Then, we calculated the F_SEF_ (see Supporting Information) and fitted the full width at half maximum (FWHM) by the Gauss function. In Figure 2e, the F_SEF_ of sample ii (Al) is 5, which is comparable to the reported value of 9 [34]. The average F_SEF_ for the sample iii–vii was calculated as 113, 355, 554, 937, and 1170, respectively, with a FWHM of ~54 nm. The maximum F_SEF_ value reached 1407 for sample vii with a thickness of ~20 μm. That is probably because as the thickness of the disordered nanofiber film increases, the probability of fluorescence undergoing multiple scattering events within the film rises. Especially for the disordered nanofibers stacked in multiple layers, each layer contributes to the scattering, and the scattered light from one layer can serve as the incident light for the next layer, leading to cumulative multiple scattering. This F_SEF_ is competitive when compared to values reported for noble metals and high-index dielectric materials, particularly considering that the refractive index of the polymer nanofibers is as low as 1.46 (Table S1).

Moreover, to exclude the enhancement effect from the Al substrate, we measured the PL intensity of R6G at the same concentration on multilayer PVAc nanofiber film transferred onto SiO_2_/Si, glass and polydimethylsiloxane (PDMS) substrates using a water-assisted floating technique. These values of PL intensity were comparable to that obtained on the aluminum substrate (Figure S4). This indicates that the fluorescence enhancement observed in the randomly oriented multilayer PVAc nanofiber film is independent of the substrate. Additionally, the free-standing films can also be transferred onto various substrates, including culture dishes, microfluidic channels, and other media on demand, making the PVAc nanofiber film suitable for a wide range of SEF applications.

### 3.3. PL Decay Dynamics and Theoretical F_SEF_

To consider the mechanism for the extraordinary F_SEF_ of PVAc nanofiber film, the theoretical F_SEF_ is given by [35]:

(1) $$F_{SEF}=\frac{\gamma_{ex}}{\gamma_{ex}^{0}}\frac{\gamma_{em}}{\gamma_{em}^{0}}\frac{\eta }{\eta^{0}}$$

where $\gamma_{ex}$, $\gamma_{em}$ and *η* are an excitation rate, emission rate, and emission collection efficiency, respectively. The superscript ‘0’ indicates the corresponding free-space quantity. Using the same optical collecting setup, *η* is equal. So, the enhancement of PL is a result of two effects in one photon process: (1) improving the excitation rate due to localized field enhancement (called excitation rate enhancement (*E_ex_*) and written as: ${E_{ex}=\gamma }_{ex}/\gamma_{ex}^{0}={\left|E\right|}^{2}/{\left|E_{0}\right|}^{2}\;$; *E* and *E*_0_ are the local electric field and incident electric field); and (2) improving the emission rate by enhancing the radiative decay rate and reducing the nonradiative decay rate, reflected both in the fluorescence lifetime and quantum yield ∅ (called emission rate enhancement (*E_em_*) and defined as: ${E_{em}=\gamma }_{em}/\gamma_{em}^{0}=\emptyset /\emptyset^{0}\;$). So, *F_SEF_* is given in a simplified form [10,36]:

(2) $$F_{SEF}=E_{ex}E_{em}=\frac{{\left|E\right|}^{2}}{{\left|E_{0}\right|}^{2}}\frac{\emptyset }{\emptyset^{0}}$$

The ∅ is the ratio of the radiative rate to the total decay rate [37]:

(3) $$\emptyset =\frac{k_{r}}{{k_{r}+k}_{nr}}$$

(4) $$\tau ={\left({k_{r}+k}_{nr}\right)}^{-1}$$

where *k_r_*, *k_nr,_* and *τ* are radiative and nonradiative decay rates and a lifetime of fluorescence molecule, respectively.

**Figure 3:**
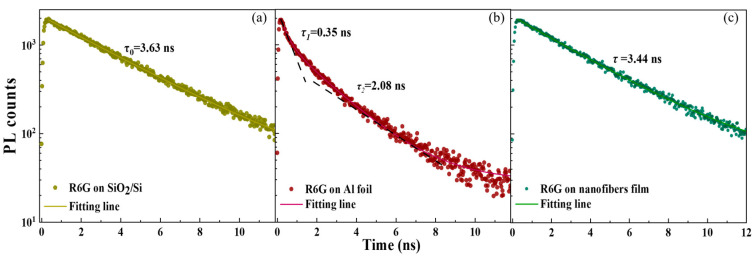
PL decay dynamics. (**a**) R6G on SiO_2_/Si substrate, serving as the intrinsic decay; (**b**) R6G on Al foil, fitting with biexponential lifetime model, consisting of a shorter lifetime of *τ*_1_ and a long lifetime of *τ*_2_; (**c**) R6G on PVAc nanofiber film.

Field enhancement ${\left|E\right|}^{2}/{\left|E_{0}\right|}^{2}\;$ of low-index PVAc produces a lower excitation rate enhancement of 3–8 (Figure 1c and Figure S1). It is not the primary factor contributing to high *F_SEF_* observed in PVAc nanofibers. Therefore, to gain further insight into ∅ and PL decay rates of R6G in the disordered PVAc nanofibers, we performed PL lifetime measurements by time-correlated single photon counting (TCSPC). Three samples were prepared shown in Figure 3**.** The intrinsic emission lifetime (τ_0_)of R6G on SiO_2_/Si was obtained from a single-exponential fit and found to be 3.63 ns (Figure 3a). Comparing the value with a lifetime of 3.79 ns and ∅ of 83% measured for R6G in water [38], we have concluded that ∅^0^ of R6G on SiO_2_/Si is 79%, as SiO_2_ is considered an insulator. The decay curve of R6G on Al displays a biexponential decay with an average lifetime (τ) of 1.79 ns (Figure 3b). The short lifetime (τ_1_) of 0.35 ns is due to the Purcell effect, while the long lifetime (τ_2_) of 2.08 ns corresponds to coupling to free space. The PL decay of R6G on PVAc nanofiber film is slow and single exponential (Figure 3c). The τ of 3.44 ns is approximate to the intrinsic lifetime of 3.63 ns. Ideally, we expect the *k_nr_* is minor and unaltered because R6G is one of the fluorescent molecules with a high quantum yield. According to the measured lifetimes, and Equations (3) and (4), we calculated the *k_r_* and ∅ of R6G on different substrates, as well as *E_ex_* and *E_em_* (Table S2). For R6G on Al, the calculated *F_SEF_* of 6.78 is comparable to the experimental *F_SEF_* of 5. But for PVAc nanofiber film, the calculated *F_SEF_* in the range of 2.85–7.60 is much lower than the experimental *F_SEF_* of 1170. This discrepancy suggests that the observed SEF in the PVAc nanofiber film is primarily mediated by other factors, such as the significant optical property of multiple scattering within the disordered nanofiber structure.

### 3.4. Random Lasing and Characteristic of Multiple Scattering

**Figure 4:**
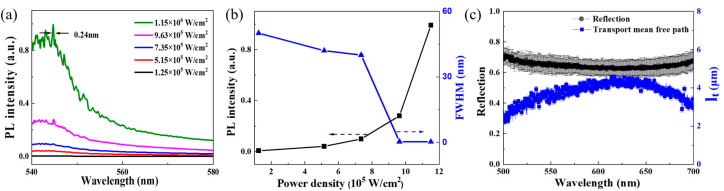
Characteristic of multiple scattering. (**a**) The random lasing was pumped at 532 nm by 10 ns pulses from 1.25 ×10^5^ W/cm^2^ to 1.15×10^6^ W/cm^2^. (**b**) The peak intensity and FWHM of the corresponding emission spectrum as a function of the pump power density. (**c**) Reflection spectra and the transport mean free path (*l_t_*) of disordered PVAc nanofiber film with a thickness of 20 μm.

In order to confirm the presence of multiple scattering effects in the disordered PVAc nanofiber film, random lasing was performed. It is well established that multiple scattering contributes to random lasers in disordered optical media [29,39]. A 10^−3^ M of R6G was used as lasing active material, drop-casting on PVAc nanofiber film. Figure 4a shows the evolution of emission spectra as the pump power density increased from 1.25 × 10^5^ W/cm^2^ to 1.15 × 10^6^ W/cm^2^. At the lower pump power density, broad emission spectra were observed, corresponding to the intrinsic fluorescence of R6G; whereas, at a pump power density of 1.15 × 10^6^ W/cm^2^, narrow and discrete peaks appeared, indicative of laser modes. The mode linewidth is 0.24 nm and the quality factor (Q=λ/FWHM) is 2270. Similar random lasing has been reported in R6G-doped polymethyl methacrylate (PMMA) nanofibers [40], and TiO_2_ nanoparticles [41]. Figure 4b presents the plots of PL intensity and FWHM as a function of pumping power density. The nonlinear variation between intensity, FWHM, and pump power density further supports the occurrence of typical random lasing, driven by multiple scattering in the disordered PVAc nanofiber film.

Further, Figure 4c shows the reflection spectra (black symbols) of PVAc nanofiber film, exhibiting a high reflectivity of about 65% in the 500–700 nm range. It implies that nanofibers with diameters comparable to the visible wavelength can serve as effective scatterers, facilitating strong visible light reflection. According to the effective diffusive medium approach, the transmission (*T*) including the multiple scattering and the ballistic contribution is given as [42]:

(5) $$T=\frac{\left(1+z_{e}\right)-\left[1+z_{e}+L/l_{t}\right]exp\;(-L/l_{s})}{L/l_{t}+2z_{e}}$$

where $z_{e}$ is the extrapolation length ratio, $z_{e}=(2/3)(1+R)/(1-R)$, R is the reflectivity, L is the thickness of the disordered material, *l_t_* and *l_s_* are the transport and scattering mean free path, respectively. The ballistic contribution $T_{B}=exp\;(-L/l_{s})$, for an opaque thick disordered medium is negligible. So, the simple form of T is written as:

(6) $$T=\frac{1+z_{e}}{L/l_{t}+2z_{e}}$$

The $l_{t}$ as a function of *R*(*λ*) could be extracted by solving Equation (6) (T=1−R, assuming negligible absorption), shown in Figure 4c (blue symbols). The *l_t_* varies between 2.5 to 4.5 μm depending on the 500 to 700 nm wavelength range. These values correspond well with the typical *l_t_* in polymer random laser (2–3 µm for visible light) [43]. In the case of anisotropic scattering, $l_{s}=l_{t}(1-g)$ where *g* is the scattering anisotropy factor and lies between 0 and 1 (0.6–0.9 as reported [44]). Thus, *l_s_* is smaller than *l_t_* and less than 2 μm, which supports random lasing in the diffusive regime ($\lambda <l_{s}<L$) [45]. The random lasing is strongly dependent on the *l_s_* of multiple scattering. A small *l_s_* means that the scatters can provide greater scattering strength [46]. Specifically, this disordered PVAc nanofiber film demonstrates desirable properties of multiple light scattering for random lasing and SEF.

### 3.5. The Limit of Detection of PVAc Nanofiber Sensor

**Figure 5:**
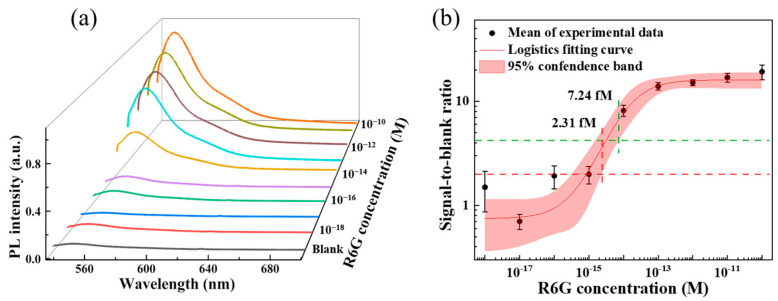
The limit of detection of PVAc nanofiber sensor. (**a**) The PL spectra of R6G with the concentration ranging from 10^−10^ M to 10^−18^ M and the blank. Each spectrum represents the average measurement from four fluorescence acquisitions. (**b**) The fluorescence SBRs vs. R6G concentration were plotted and fitted to the four-parameter logistic model (black dots show the means and error bars show the standard deviation).

The limit of detection (LoD), which refers to the smallest detectable concentration of an analyte, is one crucial performance metric for biosensors. To evaluate the LoD of PVAc nanofiber sensor, a serial dilution of R6G solution was applied dropwise on the film, forming a circular test area. The PL spectra of R6G and the blank were measured, and the results are presented in Figure 5a. A sigmoidal fluorescence signal-to-blank ratio (SBR) as a function of R6G concentration was fitted to an empirical four-parameter logistic model (Figure 5b). The experimental LoD was defined as the intersection of the lower 95% confidence interval of the sigmoidal regression with the estimated LoD [47]. We statistically estimated the LoD by $y=y_{blank}+3\sigma_{blank}$ where $y_{blank}$ and $\sigma_{blank}$ are the mean and standard deviation of the response to a blank sample (deionized water) [48]. The experimental LoD of PVAc nanofiber film is 2.31 fM. To obtain a more conservative LoD by determining 10$\sigma_{blank}$, the LoD is 7.24 fM, equivalent to 4.35 × 10^3^ molecules in 1 μL or 0.0436 molecules in a focal light spot with a diameter of 2 μm (see Supporting Information). A comparison of the reported LoD for dye sensing using noble metals and dielectric nanostructures is provided in Table S1. This low refractive index disordered PVAc nanofiber sensor demonstrates exceptional fluorescence detection capabilities compared to previously reported fluorescence biosensors. This suggests that low-index sensors like the PVAc nanofiber sensor could offer enhanced sensitivity and more cost-effective practical applications in the future.

### 3.6. Detection of Aptamer-Cy3

**Figure 6:**
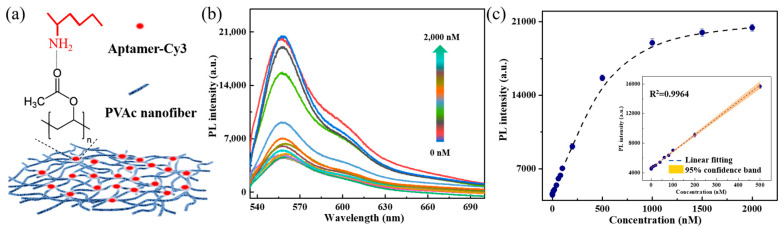
(**a**) Illustration of aptamer-Cy3 adsorbing to nanofibers; (**b**) PL spectra and (**c**) intensity of Aptamer-Cy3 with the gradient concentrations (0–2000 nM). Inset: Fitted linear relationship between PL intensity and Aptamer-Cy3 concentration (0–500 nM).

To evaluate the applicability of PVAc nanofiber sensor in biomolecule fluorescence detection, a series of different concentrations of Cy3-labeled DNA aptamer (aptamer-Cy3, Cy3-CAC TAC AGA GGT TGC GTC TGT CCC ACG TTG TCA TGG GGG GTT GGC CTG TTT TTT TTT TTG AGC CAC TGG ATA C) were deposited onto the PVAc nanofiber sensor through the hydrogen bond between the amino group (-NH_2_) of DNA and the carbonyl group (-C=O) of the PVAc nanofiber (Figure 6a). The PL spectra and intensity of aptamer-Cy3 are shown in Figure 6b,c. The PL intensity exhibits an increment as the aptamer-Cy3 concentrations, increasing from 0 to 2000 nM, and is linearly correlated with the DNA concentration within a large range from 0.1 to 500 nM; a fitting coefficient of R^2^ = 0.9964 was also obtained (inset of Figure 6c). Moreover, the LoD of aptamer-Cy3 was calculated to be 7.6 nM following the $3\sigma_{blank}/k$ formula (*k* is the slope of linear fitting). In the proof-of-concept experiment, the LoD for aptamer-Cy3 is higher than R6G, possibly because DNA molecules are not well deposited in PVAc nanofibers due to the hydrophobicity, such as aggregating on the surface of the nanofiber membrane or being washed away by water. Serving as an excellent fluorescence-enhancing sensor, this experiment verifies that PVAc nanofiber sensor can effectively detect Cy3-labeled DNA molecules in the concentration range of a few nanomoles. However, some improvements are needed in the hydrophilicity of nanofibers and the absorbability of biomolecules in future practical applications, such as adding a hydrophilic layer to the nanofibers or building stronger connections between biomolecules and nanofibers.

## 4. Conclusions

In this study, we present a fluorescence enhancement strategy based on multiple scattering within randomly oriented multilayer polymer nanofibers. This approach addresses the limitations of fluorescence enhancement techniques that typically rely on noble metals or high-index dielectric materials, as well as the weak electric field enhancement in low-index dielectric materials. The all-dielectric, low-index PVAc nanofiber sensor platform we designed demonstrates efficient fluorescence enhancement, achieving a fluorescence enhancement factor of 1170. This fluorescence enhancement is attributed to multiple scattering within the disordered multilayer nanofibers. Moreover, PL decay dynamics of the probe molecule revealed a fluorescence lifetime of 3.44 ns in the sensor, which is almost identical to its intrinsic lifetime of 3.63 ns. This indicates that the radiative rate and PL quantum efficiency contribute minimally to the fluorescence enhancement in the PVAc nanofiber sensor. And the sensor preserves the intrinsic properties of the probe molecule, an important feature for biosensors to ensure the analyte remains unaltered. In combination with the weak electric field enhancement observed in the PVAc nanofibers (ranging from 3 to 8), this fluorescence enhancement diverges from traditional approaches that rely on enhancing the radiative rate and the electric field. The underlying mechanism of multiple scattering was further validated through random lasing experiments, which revealed a laser mode with a linewidth of 0.24 nm and a quality factor of 2270. From the reflectance spectrum, the $l_{t}$ was estimated to be in the range of 2.5–4.5 μm, while the $l_{s}$ was less than 2 μm. These values align with typical characteristics observed in low-index dielectric nanostructures exhibiting random lasing and confirm that the disordered PVAc nanofiber film possesses the desired properties for effective light scattering as an optical sensor. Based on these characteristics, the LoD for the R6G reached 7.24 fM, and for Cy3-labeled DNA aptamer, it reached 7.6 nM with a large linear range of 0.1–500 nM, exhibiting the potential of this polymer nanofiber sensor for advancing nondestructive detection in biosensing applications.

Furthermore, the electrospinning process for preparing PVAc nanofibers is significantly more straightforward for mass production, presenting an attractive prospect for low-cost and high-performance fluorescent sensor applications. The PVAc acts as a representative polymer but is not limited to this; however, it can be other low-index materials that can be fabricated into disordered nanostructures with multiple scattering. Consequently, this research will promote the development and application of all-dielectric, low-index nanostructure-based multiple scattering-enhanced fluorescence sensors.

## Data Availability

Data are contained within the article or supplementary material.

## Supplementary Materials

The following supporting information can be downloaded at: https://www.mdpi.com/article/10.3390/bios15020097/s1, Figure S1: The FDTD simulated electric field intensity distribution on the cross section of nanofibers with different arrangement structures for incident plane waves at 532 nm and nanofibers with the refractive index of 1.46. Figure S2: As-prepared PVAc nanofibers film on aluminum foil, SEM, diameter distribution with the average diameter of 550 nm. Figure S3: The optical images of sample i–vii. Figure S4: Photographs of a free-standing PVAc nanofiber film floating on the water; the PL intensity of R6G with the same concentration on PVAc nanofiber film transferred onto Al foil, SiO_2_/Si, glass, and PDMS, respectively. Table S1 [17,19,20,23,49,50,51,52,53,54,55,56,57,58,59,60]: An overview of typical materials and structure, enhancement mechanism, fluorescence probe/analytes (F/A), fluorescence quantum yields (∅), and the max of F_SEF_, LoD. Table S2: Lifetime measurements of R6G on the SiO_2_/Si substrate, Al foil, and the PVAc nanofiber film. The calculated values of lifetime, radiative rate, quantum yield, emission rate enhancement (E_em_ =$\;\emptyset /\emptyset^{0}$), excitation rate enhancement (E_ex_ = |E|^2^/|E_0_|^2^), calculated PL enhancement factor (E_ex_ · E_em_), and experimental F_SE_; The calculation of the LoD, and the number of probe molecules in a focal light spot and F_SEF_.
