# Supplementary material for: Multiple Scattering-Enhanced Fluorescence Within Randomly Oriented Low-Index Polymer Nanofiber Sensors
Source: Biosensors (Basel). 2025 Feb 8;15(2):97. doi: 10.3390/bios15020097 (PMC11853261; doi:10.3390/bios15020097)
Supplement: Supplementary file 1 [file biosensors-15-00097-s001.zip › biosensors-3454588-supplementary.pdf]

Supporting information

Multiple Scattering-enhanced Fluorescence within Randomly Oriented Low-Index Polymer Nanofibers Sensor

Jing Sun [1, 2], Tao Huang [3, *] and Zhongyang Wang [1, 2, *]

[1] Shanghai Advanced Research Institute, Chinese Academy of Sciences, Shanghai 201210, China; sunj@sari.ac.cn
[2] University of Chinese Academy of Sciences, Beijing 100049, China;
[3] Department of Materials Science and Engineering, Southern University of Science and Technology, Shenzhen 518055, China;
* Correspondence: huangt@sustech.edu.cn (T. H); wangzy@sari.ac.cn (Z. W)

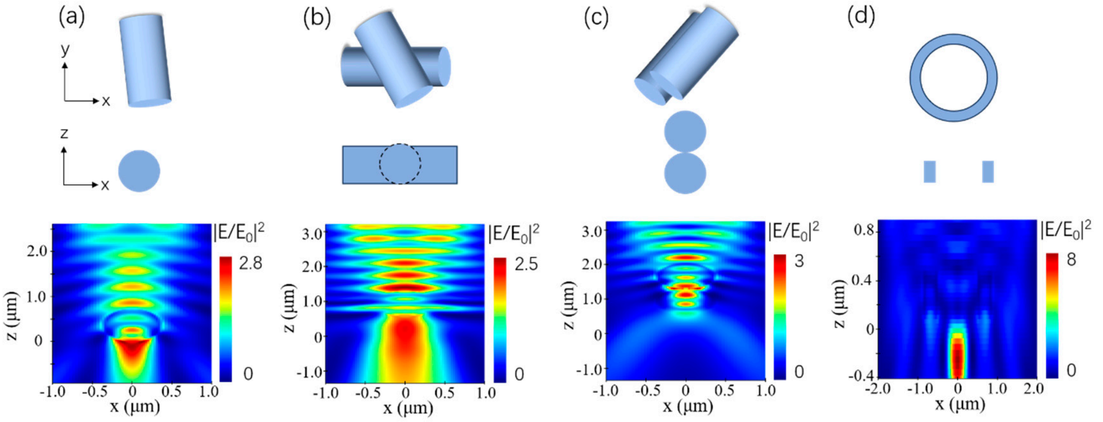

Figure S1. The FDTD simulated electric field intensity distribution on the cross section of single fiber (a), two crossed nanofibers (b), vertical parallel nanofibers (c), and ring nanofibers (d) for incident plane waves at 532 nm and nanofibers with the refractive index of 1.46.

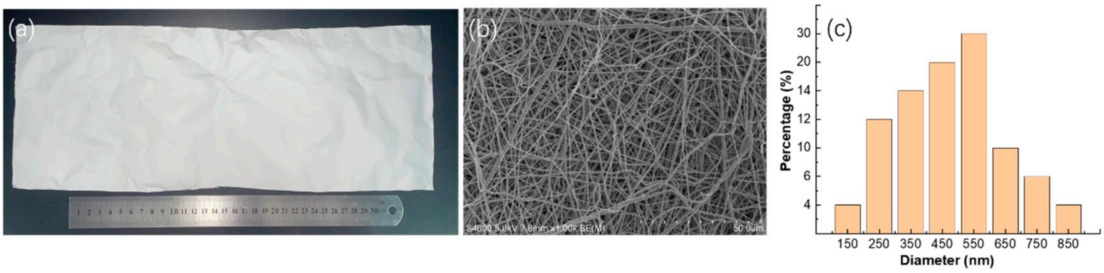

Figure S2. (a) As prepared PVAc nanofibers film on aluminum foil; (b) Scanning electron micrographs (SEM) of polymer nanofiber film; (c) Nanofibers diameter distribution and the average diameter of 550 nm.

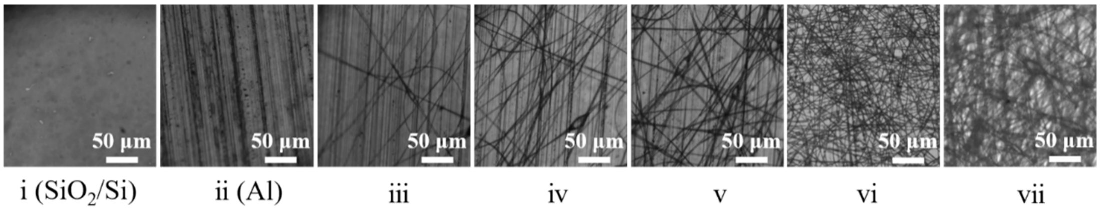

Figure S3. The optical images of i (SiO_2/Si), ii (Al foil), nanofibers with increasing density and thickness, iii (a few nanofibers), iv (dozens of nanofibers), v (layer of nanofibers), vi (a few layers), vii (dozens of layers with a thickness of ~20 µm).

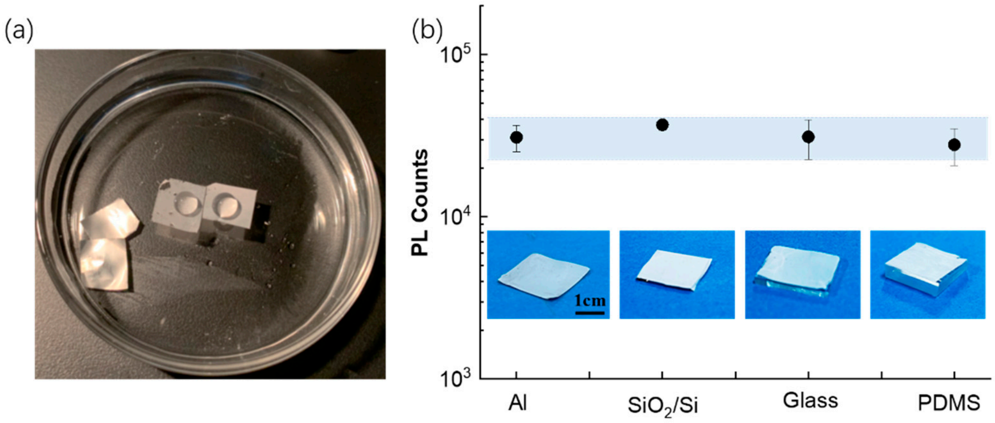

Figure S4. (a) Photographs of a free-standing PVAc nanofiber film floated on the water; (b) The PL intensity of R6G with the same concentration on PVAc nanofibers film, which is transferred on Al foil, SiO_2/Si, glass, polydimethylsiloxane (PDMS), respectively.

Table S1. Overview of typical materials and structure, enhancement mechanism, fluorescence probe/analytes (F/A), fluorescence quantum yields (∅), the max of enhancement factors (F_{SEF}), limit of detection (LoD).

Nanostructure	enhancement mechanism	F/A	∅	F_{SEF}	LoD	Ref.
Au bowtie	LSPR	TPQDI	0.025	1340	single molecule	[45]
Single Au Nanorod	LSPR	NDI-2TEG-3T	1.3×10^{-4}	10^4	single molecule	[46]
Au@Ag nanospheres	LSPR	Cy5-DNA	NA	100	3.1 pM	[47]
Ag nanoparticles	Plasma	R6G	0.9	20.5	NA	[48]
		Fluorescein	0.95	100		
		Acridine orange	0.29	8.34		
		Rhodamine B	0.41	5.13		
		Bosin-Y	0.32	4.3		
Au nanorods dimer	LSPR	Cy7- DNA origami	0.28	1600	single molecule	[49]
Si nanopellet array	higher-order magnetic resonance	HEX-DNA	NA	~10	1.37 fM	[50]
B-/P codoped single c-Si nanosphere	dipole Mie resonance	Rhodamine B	>0.3	200	NA	[51]
Si_3N_4 nanohole	bound states in the continuum	R6G	NA	790	NA	[52]
Si dimer	EM modes	crystal violet	NA	270	NA	[17]
Si dimer	EM hot spots	Nile Red	NA	1900	NA	[19]
Si nanocolumn array	EM resonance	TPE-4TA-HSA	NA	10	18.75 ng mL^{-1}	[53]
Si nanorod metasurface	EM resonance	AF555-IgG	NA	~10	5 pg/mL (34 fM)	[54]
Porous TiO_2 layer (n=2.6)	EM field enhancement	crystal violet	NA	500	NA	[55]
Nanogap-rich TiO2 film	EM field enhancement	crystal violet	NA	2000	NA	[23]
ZnO nanowires (n=2.0)	NA	FITC-IgG/ AFP	NA	20	1 pg/mL	[20]
Sharp ZnO nanowires	NA	Alexa 546 - streptavidin	NA	11	417 fM	[56]
Polymer nanofiber film (n=1.46)	multiple scattering	R6G	0.79	1400	7.24 fM	This work
		Cy3-DNA	/	/	7.6 nM	

LSPR: Localized Surface Plasmon Resonance; EM: electric-magnetic; TPQDI: N,N′-bis(2,6-

diisopropylphenyl)-1,6,11,16-tetra-[4-(1,1,3,3-tetramethylbutyl)phenoxy]quaterrylene-3,4:13,14-bis(dicarboximide); Cy5(3/7): Cyanine 5(3/7); HSA: Human serum albumin; TPE-4TA: tetrazolate-tagged tetraphenylethylene derivative; IgG: immunoglobulin G; FITC: Fluorescein-5-isothiocyanate; AFP: human α-fetoprotein; NDI-2TEG-3T: Naphthalene diimide-terthiophene derivative.
NA: No available.

Table S2. Lifetime measurements of R6G on SiO_2/Si substrate, Al foil and PVAc nanofiber film. And the calculated values of Lifetime, radiative rate, quantum yield, emission rate enhancement ($E_{em}=\emptyset/\emptyset^0$), excitation rate enhancement ($E_{ex}=|E|^2/|E_0|^2$), calculated PL enhancement factor ($E_{ex} \cdot E_{em}$), and experimental PL enhancement factor (F_{SEF}).

	SiO_2/Si	Al foil	PVAc nanofiber film
τ_1/ns	/	0.35	/
τ_2/ns	/	2.08	/
τ/ns	3.63	1.79	3.44
k_r/s^{-1}	2.17×10^8	5×10^8	2.18×10^8
$\emptyset$	0.79	0.895	0.75
$E_{em} = \emptyset/\emptyset^0$	/	1.13	0.95
$E_{ex} = |E|^2/|E_0|^2$	/	~6	~3–8
Calculated F_{SEF} ($E_{ex} \cdot E_{em}$)	/	6.78	2.85–7.60
Max experimental F_{SEF}	/	7.1	1407
Average experimental F_{SEF}		5	1170

***Calculation* of the *LoD*:**

For the sigmoidal analysis of fluorescence intensity signal-to-blank ratios (SBRs), a four-parameter logistic function was fitted:

$$y = A_2 + \frac{A_1 - A_2}{1 + (x/x_0)^P}$$

Where, A_1 and A_2 set the upper and lower limit of the function, respectively, x_0 is the center, and p is the power. Fitting was performed in Origin using nonlinear curve fit functions for logistic fit.

According to International Union of Pure and Applied Chemistry (IUPAC), a typical approach to estimate LoD:

$$y = y_{blank} + 3\sigma_{blank}$$

Where, y_{blank} and σ_{blank} are the mean and standard deviation of the response to a blank sample (deionized water).

To obtain a more conservative LoD, variation of estimate LoD:

$$y = y_{blank} + 10\sigma_{blank}$$

Back-calculated concentrations were determined using the "Find X from Y" feature in origin.

The number of probe molecules in a focal light spot:

For the LoD of 7.24 fM, the number of probe molecules in a focal light spot (N) was calculated:

$$N = \frac{V \times \text{LoD} \times N_A}{S_{pm}} \times S_{fs}$$

Where, V is solution volume of probe molecules. N_A is Avogadro constant. S_{pm} is the area formed by the probe molecule of 3.14 mm^2. The diameter (d) of focal light spot of the microscope objective is 2 μm, S_{fs} is the area of focal light spot.

$$N = (10\ \mu L \times 7.24\ fM \times 6.02 \times 10^{23}\ M^{-1}/3.14\ mm^2) \times 3.14 \times 10^{-6} mm^2 = 0.0436.$$

Calculation of the Surface fluorescence enhancement factor (F_{SEF}):

For the calculation, the under identical experimental conditions measured fluorescence spectra of all samples (the number of probe molecular, laser power, collection time, etc). The F_{SEF} was calculated according to the formula [**Error! Bookmark not defined.**]:

$$F_{SEF} = \frac{I_{SEF} - I_{SEF\_b}}{I_{con} - I_{con\_b}}$$

Where the I_{SEF} and I_{con} are the integral intensities of fluorescence spectrum of R6G on the PVAc nanofiber film and the control sample of SiO_2/Si, respectively. I_{SEF_b} and I_{con_b} are the corresponding integral intensities of the background spectrum with deionized water, respectively.
